# Function of Dental Follicle Progenitor/Stem Cells and Their Potential in Regenerative Medicine: From Mechanisms to Applications

**DOI:** 10.3390/biom11070997

**Published:** 2021-07-07

**Authors:** Ruiye Bi, Ping Lyu, Yiming Song, Peiran Li, Dongzhe Song, Chen Cui, Yi Fan

**Affiliations:** 1 National Clinical Research Center for Oral Diseases, State Key Laboratory of Oral Diseases, Department of Oral and Maxillofacial Surgery, West China Hospital of Stomatology, Sichuan University, Chengdu 610041, China; david-bry@163.com (R.B.); 2014181641012@stu.scu.edu.cn (P.L.); 2 National Clinical Research Center for Oral Diseases, State Key Laboratory of Oral Diseases, Department of Cariology and Endodontics, West China Hospital of Stomatology, Sichuan University, Chengdu 610041, China; doudouko10@gmail.com (P.L.); simitater@outlook.com (Y.S.); dongzhesong@scu.edu.cn (D.S.); 3 Guangdong Province Key Laboratory of Stomatology, Hospital of Stomatology, Guanghua School of Stomatology, Sun Yat-Sen University, Guangzhou 510055, China

**Keywords:** tooth development, differentiation, tissue regeneration, stem cell therapy, scaffold, immunoregulation

## Abstract

Dental follicle progenitor/stem cells (DFPCs) are a group of dental mesenchyme stem cells that lie in the dental follicle and play a critical role in tooth development and maintaining function. Originating from neural crest, DFPCs harbor a multipotential differentiation capacity. More importantly, they have superiorities, including the easy accessibility and abundant sources, active self-renewal ability and noncontroversial sources compared with other stem cells, making them an attractive candidate in the field of tissue engineering. Recent advances highlight the excellent properties of DFPCs in regeneration of orofacial tissues, including alveolar bone repair, periodontium regeneration and bio-root complex formation. Furthermore, they play a unique role in maintaining a favorable microenvironment for stem cells, immunomodulation and nervous related tissue regeneration. This review is intended to summarize the current knowledge of DFPCs, including their stem cell properties, physiological functions and clinical application potential. A deep understanding of DFPCs can thus inspire novel perspectives in regenerative medicine in the future.

## 1. Introduction

Dental follicle (DF) is a loose connective tissue surrounding the dental papilla and enamel organ during the early developing stages of the tooth [1]. It is an ectomesenchyme-derived tissue originating from cranial neural crest and is a crucial participant during tooth development. DF can give rise to periodontium tissue, including cementum, alveolar bone and periodontal ligament (PDL) [2]. The formation of periodontal attachment apparatus is crucial for the building of root-bone interface during root development process. Moreover, DF plays a central role in the regulatory network during the tooth eruption process by regulating alveolar bone resorption and formation [3,4]. As research focusing on DF goes deeper, a mesenchymal stem cells (MSCs) population residing in DF receives growing attention. Dental follicle cells were first isolated from rat molars characterized by a fibroblast-like appearance in 1992 [5]. Later, a population of colony-forming and plastic adherent cells were isolated from DF of human third molars in 2005 [6]. Since these cells harbor properties similar to the classic MSCs, they are defined as dental follicle progenitor/stem cells (DFPCs), a novel kind of dental MSCs. Human DFPCs (hDFPCs) express a series of classic MSC markers, including CD44, CD73, CD90, CD105, NOTCH-1, NESTIN and STRO-1, while negative for hematopoietic stem cell markers such as CD34, CD45 and CD11b [7,8,9,10].

DFPCs are capable of multipotent differentiation with high pluripotency. A series of pluripotent genes, including Octamer-binding transcription factor 4 (OCT-4), sex determining region Y box-2 (SOX-2) and NANOG, were shown to be expressed in DFPCs, which confirmed their self-renewal capacity and multipotent potential [11,12]. The heterogeneity of DFPCs was revealed by the cloning of three distinct DF cell lines with unique characteristics [13]. The first line was highly proliferative without mineralization behavior, indicating that it might contribute to PDL-type lineage. The second line had extreme high alkaline phosphatase (ALP) activity, suggesting an undifferentiated state. The third cell line might give rise to cementoblasts or alveolar bone osteoblastic lineage for their mineralization feature [13]. This profound cellular heterogeneity of DFPCs could explain their multi-differentiation potential. Resulting from the neural crest origin, DFPCs can differentiate into osteoblasts, adipocytes, chondrocytes, cementoblasts and periodontal ligament cells as well as neuronal cells [14,15]. Therefore, DFPCs are regarded as a promising candidate for regenerative medicine and tissue engineering. Compared with MSCs of other origins, DFPCs have several advantages in regard to tissue regeneration. First, DFPCs can be separated from impacted third molars. The extraction of the third molar is harmless to normal dentition and minimally invasive, which makes DFPCs easily accessed. Second, DFPCs are a kind of adult stem cells obtained from developing tissue. They may exhibit greater multilineage differentiation potential. Third, studies showed that DFPCs had higher proliferation ability compared with dental pulp stem cells (DPSCs) [16]. Research highlighted that DFPCs possessed more similar protein profiles to cranial neural crest cells (CNCCs) compared with DPSCs [17]. Furthermore, it is feasible to cryopreserve DF tissue as a resource for DFPCs in the long run [18]. These features increase the potential application of DFPCs in tissue engineering, especially in the orofacial region.

In this review, we aim to summarize the current knowledge of DFPCs focusing on their function in tooth development, regulatory mechanisms of the multilineage differentiation potential together with the application in stem cell-based regenerative medicine and tissue engineering.

## 2. The Role of DFPCs during Tooth Eruption

Tooth eruption is a biological process whereby a tooth within bone emerges into the oral cavity and reaches the occlusal plane to properly function. This process can be divided into three stages, the pre-eruptive, eruptive and post eruptive tooth movement [19]. During the pre-eruptive tooth movement stage, dental epithelial cells develop into enamel organ and recruit dental mesenchyme inside the tooth bud. The mesenchyme originated cells gather into dental papilla located apically to the enamel organ and the surrounding DF. The enamel organ originated ameloblasts secrete enamel while dental papilla originated odontoblasts produce dentin. This process continues until the tooth crown is gradually formed and ready to emerge. The eruptive stage starts from the initiation of tooth root development and lasts until the crown emerges and reaches the occlusal plane. This process is subdivided into intraosseous and supraosseous phases, which requires polarized resorption and formation of alveolar bone surrounding the tooth to remove the coronal resistance and provide apical motivation at the same time. The main biological activity during post-eruptive tooth movement stage is the maturation and stabilization of periodontium tissue to achieve proper function.

During the tooth eruption process, DF plays a crucial role in providing the traction power and forming the eruption pathway. Surgical removal of DF prevented tooth eruption, which proved that DF exerted great influence on the eruption process, especially during the second and third stage [3]. As the tooth germ gradually develops, DF can be separated into a coronal part and a periapical/basal part. The former is close to the tooth crown and develops into periodontium, while the latter is located apically from the dental papilla [20]. To elucidate the diverse function of DF, an elegant study selectively eliminated the coronal or basal part of DF, which led to arrested tooth eruption under either circumstance [21]. Removal of the coronal part of DF interrupted coronal bone resorption and the formation of the eruption pathway while absence of the basal part of DF led to the loss of apical bone formation [21]. This study revealed that coronal cortical shell resorption and apical bone formation during tooth eruption are regulated by two adjacent parts of DF respectively.

Root development and the establishment of root-bone connection are closely intertwined with tooth eruption [22]. The constitution of functional tooth-bone interface (TBI) requires proper formation of the acellular cementum, PDL and cryptal bone. DFPCs are responsible for the building of TBI, which makes them the major contributors to alveolar bone remodeling and periodontium tissue development. DFPCs are able to differentiate into osteoblasts to participate in alveolar bone formation while exerting regulatory influence on monocyte/osteoclast lineage differentiation and function [23,24]. Recent studies discovered that spatiotemporal gene expression of DFPCs are key to regulating asymmetric bone resorption and bone formation around bony crypt during tooth eruption [25,26].

### 2.1. Regulation of Osteoclastogenesis and Bone Resorption Process

Mononuclear cells (osteoclast precursors) and osteoclasts are essential to the formation of the eruption path [27]. Tartrate-resistant acid phosphatase (TRAP, a marker enzyme of osteoclast lineage) positive mononuclear cells were detected in DF prior to eruption and then gradually reduced during the eruption process [28]. Studies show a colony of mononuclear cells swarming into the DF at a specific time prior to eruption [28,29]. These osteoclast precursors are derived from hematopoietic stem cells and differentiated from monocyte-macrophage lineage in bone marrow or peripheral blood [30]. Recently, research discovered that various adult tissue-resident macrophages originate from erythron-myeloid progenitors (EMPs) during embryonic stage [31]. This finding suggests a novel source of osteoclasts in the orofacial region, which may participate in the tooth eruption.

Osteoclastogenesis required for eruption is tightly controlled by different pathways and cytokines, such as colony-stimulating factor-one (CSF-1), receptor activator of nuclear factor kappa (RANK)-RANK ligand (RANKL)-osteoprotegerin (OPG) axis and parathyroid hormone related peptide (PTHrP). These factors function directly or in crosstalk to form a regulatory network for bone resorption during tooth eruption. CSF-1, also named as macrophage colony-stimulating factor (M-CSF), is a prerequisite factor for proliferation and differentiation of osteoclast precursors [32,33]. It is reported that DFPCs expressed chemokines for mononuclear cells, CSF-1 and monocyte chemotactic protein-1 (MCP-1), to recruit monocytes from the peripheral blood before tooth eruption [12]. Failure of tooth eruption occurred in CSF-1 knockout mice due to impaired osteoclast formation and bone resorption [34]. Endothelial monocyte-activating polypeptide-2 (EMAP-2) expressed in DF, also has a chemotactic effect on mononuclear cells by upregulating the expression of CSF-1 and MCP-1. CSF-1 and EMAP-2 downregulate secreted frizzled-related protein-1 (SFRP-1), a cytokine that suppresses osteoclastogenesis, to promote osteoclast formation [35]. Moreover, the stellate reticulum layer of enamel organ can release transforming growth factor β1 (TGF-β1) and interleukin-1α (IL-1α) to enhance CSF-1 expression in DF through a paracrine manner [36,37,38,39]. These findings reveal an epithelial-mesenchymal interaction to stimulate osteoclast formation through DF.

CSF-1 can also activate osteoclastogenesis by upregulating the expression of RANKL and downregulating OPG [40,41]. RANKL/RANK/OPG axis is indispensable for osteoclast differentiation and bone resorption [42]. Briefly, the transmembrane or secreted RANKL combines with the RANK on the osteoclast precursor cells and thereby contributes to differentiation, survival, fusion and activation of osteoclasts. On the contrary, OPG is a secreted decoy receptor that competitively binds to RANKL to inhibit RANK activation and bone resorption [33]. A recent study has demonstrated significantly higher expression of RANKL in the coronal part of human DF compared with the apical part, suggesting the enhancement of osteoclastogenesis in the coronal bone shell [25]. This activation of RANKL/RANK signaling leads to an expansion of the TBI [43]. It is reported that non-canonical Wnt ligand Wnt5a abundantly upregulated RANKL expression in DFPCs [24]. Moreover, vascular endothelial growth factor (VEGF) is another key factor highly expressed in DF, which stimulates the expression of RANK in osteoclast precursors [44].

The signaling crosstalk mechanisms centered on RANKL/RANK/OPG axis are gradually unraveled. Delayed tooth eruption occurs in patients with cleidocranial dysplasia (CCD), a runt-related transcription factor 2 (Runx2) mutation/haploinsufficiency related disease. DFPCs isolated from CCD patients displayed significantly lower osteoclast-inductive ability [45,46]. This resulted from lower ratios of RANKL/OPG and RANKL/RANK regulated by abnormal RUNX2-miR-31-special AT-rich sequence-binding protein 2 (SATB2) loop. Reduced RUNX2 (a transcriptional inhibitor of miR-31), higher miR-31 and downregulated SATB2 disturbed the osteoclast-inductive signaling in DFPCs of CCD patients [47].

Parathyroid hormone-related peptide (PTHrP) is another key factor responsible for osteoclast formation [48]. It has been demonstrated that PTHrP-null mice displayed a failure of tooth eruption with normal developed tooth trapped in bone, since no formation of eruption pathway took place [48]. In situ hybridization showed highly expressed PTHrP in the coronal epithelial layers while its receptor, parathyroid hormone 1 receptor (PTH1R), was positive in the surrounding alveolar bone and DF [48]. A coculture experiment confirmed that epithelial-original PTHrP could stimulate DFPCs to induce osteoclast differentiation and contribute to coronal bone resorption [49]. PTHrP functions to stimulate the expression of RANKL while downregulating OPG in dental follicle cells [50,51].

Furthermore, gap-junction communication plays a significant role during tooth eruption since its major subunit, gap-junction protein, alpha 1, 43 kDa (CX43), is a highly expressed molecule in human DF [25]. CX43 and gap-channels are reported to be necessary for the development and bone resorption activities of osteoclast precursors and mature osteoclasts [52,53,54]. It is also involved in osteoblast differentiation, formation and mineralization of the bone matrix [52,55]. The detailed regulatory mechanisms of gap-junction communication in DFPCs require further investigation.

### 2.2. DFPCs Contribute to Osteoblast Differentiation and Bone Formation

#### 2.2.1. DFPCs Are Involved in Apically Alveolar Bone Formation In Vivo

**Figure 1:**
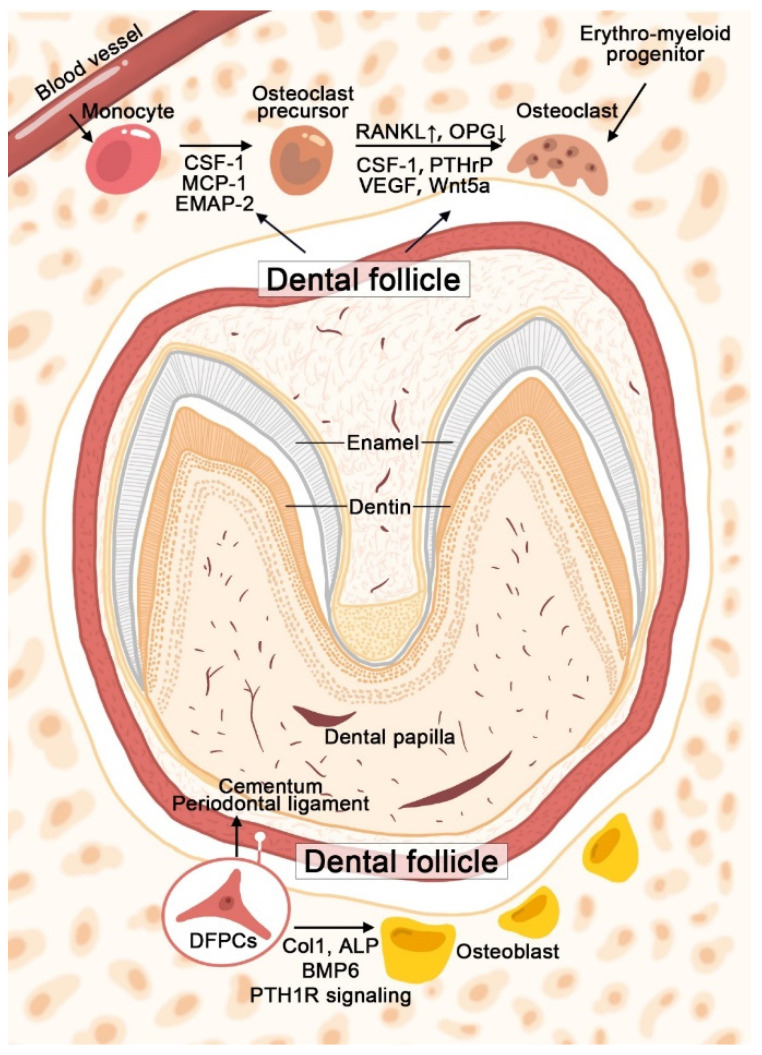
Schematic graph illustrates the mechanisms of DF/DFPCs in regulating osteoclast formation and osteoblast differentiation during tooth eruption. Colony-stimulating factor-one (CSF-1), monocyte chemotactic protein-1 (MCP-1), endothelial monocyte-activating polypeptide-2 (EMAP-2), receptor activator of nuclear factor kappa ligand (RANKL), osteoprotegerin (OPG), parathyroid hormone related peptide (PTHrP), vascular endothelial growth factor (VEGF), collagen type 1 (Col1), alkaline phosphatase (ALP), bone morphogenetic protein 6 (BMP6) and parathyroid hormone 1 receptor (PTH1R).

Peri-apical alveolar bone formation of developing tooth has long been proposed as a critical factor for eruption. It is reported that bone formation started to fill the alveolar crypt of rat first molars at the postnatal day 10 and lasted through the eruption process [56,57]. High levels of osteogenic-related factors, such as collagen type 1 (COL1) and ALP, were expressed in human DF, indicating its participation in bone formation [25]. Research showed that bone morphogenetic protein 6 (BMP6) was expressed predominately in osteoblasts within the base of alveolar bony crypt [58]. After using siRNA to interfere with the BMP6 expression in rat dental follicles, alveolar bone formation was significantly reduced, resulting in delayed or failure of eruption [58]. Therefore, the increased expression of BMP6 observed in the DF tissue may reflect the activation and maintenance of the osteogenesis ability of DFPCs to provide the motive force during eruption [59]. The development of distinct reporter mice and lineage tracing methods boost research focusing on tissue development. Through the generation of lineage-tracing models, Osterix (Osx), PTHrP, GLI family zinc finger 1 (Gli1) and Prx1 positive MSCs were found residing in DF, which can be defined as different DFPCs populations [60,61,62,63]. Gli1Cre^ER^ and Prx1Cre mouse models were used to identify MSCs while OsxCre is widely accepted as a promoter targeting osteoblast precursors. Conditional knockout of PTH1R in Osx, Prx1 or PTHrP positive cells resulted in failure of tooth eruption, indicating the significance of PTH1R signaling during this process [60,61,63]. It is shown that Prx1 and PTHrP expressing DFPCs could differentiate into osteoblasts located in the alveolar bone crypt [61,63]. The use of PTHrPCre^ER^ system to eliminate PTH1R in DFPCs revealed the autocrine manner of PTHrP to regulate bone formation. It is reported that autocrine PTHrP could enhance osteogenic differentiation of DFPCs independently of the hedgehog signaling pathway [64]. Moreover, ablation of PTH1R using Prx1Cre led to reduced alveolar bone volume, which mainly resulted from impaired bone formation since bone resorption was not affected [63]. However, Prx1 is predominantly expressed in orofacial bone-marrow-derived mesenchymal stem cells (OMSCs). To what extent DFPCs contribute to the observed phenotype requires further investigation. Figure 1 illustrates the brief mechanisms of DF/DFPCs regulating osteoclast formation and osteoblast differentiation during tooth eruption.

#### 2.2.2. DFPCs Differentiate into Osteogenic Lineage In Vitro

Considering the multipotent potential of DFPCs, a large number of studies focused on the regulatory mechanisms of the osteogenic differentiation of DFPCs. Researchers have speculated that the molecular mechanisms in DFPCs during osteogenic differentiation are distinct from that of bone marrow-derived MSCs (BMMSCs) [65]. Unlike BMMSCs, the expressions of Runx2, distal-less homeobox 5 (DLX-5) and msh homeobox 2 (MSX-2) of DFPCs were unaffected during osteogenic induction in vitro while the trend of DLX-3 was in consistent with BMMSCs. Various factors and pathways are involved in the regulatory network of osteogenesis, the top hits among which include BMP signaling pathway, Wnt signaling pathway and transforming growth factor (TGF) signaling pathway as well as their crosstalk and interaction.

BMP2 contributes to the osteogenic differentiation of DFPCs by inducing Patched 1 (PTCH1), suppressor of fused (SUFU) and PTHrP to repress hedgehog signaling pathway under osteogenic induction [66,67]. BMP2 and its downstream transcription factor DLX3 form a positive-feedback loop of the BMP2-dependent SMAD signaling pathway to direct osteogenic differentiation of DFPCs [66]. BMP2 also interacts with the WNT/β-catenin pathway by stimulating β-catenin phosphorylation via protein kinase A (PKA). BMP2 can facilitate LEF1/SMAD4/β-catenin complex to bind with DLX3 promoter to enhance osteogenesis. In a positive-feedback manner, β-catenin also activates PKA to sustain BMP2/DLX3-mediated osteogenesis of DFPCs [66].

Due to the potent osteogenic ability of BMP9, transfecting DFPCs with BMP9 resulted in significant enhancement of ALP expression and calcium deposition, which was dependent on the downstream mitogen-activated protein kinases (MAPK) signaling pathway [68]. Another study using BMP9 transfected DFPCs to investigate the osteogenic activities under inflammation microenvironment stimulated by TNF-α. TNF-α activated canonical Wnt signaling pathway and suppressed the non-canonical pathway, leading to the downregulation of osteogenic ability. However, the addition of Dickkopf 1 (DKK1), a canonical Wnt signaling inhibitor, also led to decreased osteogenesis. The inhibition effect was reduced when DKK1 was added with TNF-α, suggesting a possible approach for the treatment of alveolar bone defects resulting from periodontitis [69].

In addition to the study focusing on interaction between BMP and Wnt signaling, PKC/Akt axis, naked cuticle homolog 2 (Nkd2), adenomatosis polyposis coli down-regulated 1 (APCDD1) and long noncoding RNA maternally expressed 3 (lncRNA MEG3) also function through Wnt signaling pathway. Classical PKCs are reported to inhibit the osteogenesis ability of DFPCs by regulating canonical Wnt signaling and the expression of β-catenin together with Akt [70]. It is reported that Nkd2, a signal-inducible feedback antagonist of the canonical Wnt signaling, promotes the osteogenic differentiation of DFPCs through Wnt/β-catenin signaling [71]. Another Wnt inhibitor, APCDD1, stimulates osteogenesis by increasing the expression of β-catenin and osteogenic markers in DFPCs [72]. Lnc RNA MEG3 and its downstream are involved in gene silencing. It was found that MEG3 significantly decreased in DFPCs, which stimulated the Wnt/β-catenin pathway via activating Wnt gene promotors [73]. These investigations provide novel mechanisms in regulating osteogenesis of DFPCs.

Another well-studied osteogenic-related pathway is TGF signaling. Inflammation microenvironment caused by lipopolysaccharide (LPS) suppressed osteogenesis of DFPCs. LPS-treated DFPCs showed low level of TGF-β1 and high level of TGF-β2 while inhibition of TGF-β2 improved expression of TGF-β1 and osteogenic ability. Thus, TGF-β2 can be a promising target in treatment of inflammation related alveolar bone loss [74]. It has been reported that the injection of bleomycin into DF inhibited tooth eruption, which is related to decreased osteogenic capacity and increased TGF-β1 expression. SMAD7 is a crucial mediator in the downstream cascade of TGF signaling by suppressing Runx2 expression in DFPCs [75].

Besides, there are also other effective regulators involved in osteogenic differentiation of DFPCs. It is demonstrated that nuclear factor I-C (NFIC) stimulated osteogenesis of DFPCs by increasing ALP activity and the expression of osteogenic-related cytokines including Runx2, Col1 and β-catenin [76]. Another signaling pathway is AMP-activated protein kinase (AMPK) and its downstream cascade, which is crucial in cellular energy and metabolic homeostasis [77]. It is shown that activation of AMPK decreased ALP activity and the expression of osteogenic markers in short term cultures. DFPCs are sensitive to AMPK in early stages of osteogenic differentiation and this process is related to autophagic activity.

The majority of cell studies in vitro were performed in static conditions, while Salgado et al. explored the influence of dynamic culture by innovatively applying centrifugal force on DFPCs or DPSCs laden porous 3D scaffolds. Results showed that dynamic conditions not only promoted the proliferation of DFPCs, but also increased osteogenic-related gene expressions, ALP activity and osteopontin (OPN) deposition compared with static culture conditions. Interestingly, the dynamic conditions exerted distinct effects on DPSCs, including lower ALP activity and OPN secretion [78]. Thus, the effects of different culture conditions on cells from various sources should be explored and chosen wisely according to the final tissue engineering target and clinical application.

### 2.3. DFPCs Give Rise to Periodontal Attachment Apparatus

Root development requires epithelial stimulation from Hertwig’s epithelial root sheath (HERS), a bilayer structure developed from the inner and outer enamel epithelium. This epithelial–mesenchymal interaction induced DFPCs to differentiate into cementoblasts [79,80,81]. The expression of Wnt3a, a representative canonical Wnt ligand, was detected in HERS during root formation. Wnt3a stimulated the expression of Runx2, Osx and Alp in DFPCs, indicating that early cementoblast/osteoblast differentiation of DFPCs was induced through Osx and canonical Wnt signaling [82].

Ablation of PTH1R in Osx^+^ DFPCs using conditional knockout mouse model led to root ankylosis characterized by the absence of PDL, abnormal cellular cementum formation along with accelerated cementoblasts differentiation [60]. Histone deacetylace-4 (HDAC4) may be a key downstream mediator during this process since the ablation of HDAC4 partially recapitulated the phenotypes, including thicker cementum. PTH1R deficiency in PTHrP expressing DFPCs also resulted in the loss of periodontal attachment and unusual formation of cellular cementum [61]. Knockout of PTH1R shifts the cell fate of DFPCs to non-physiological cementogenesis. The cementogenesis ability of DFPCs is driven by BMPs produced by HERS and enamel matrix derivatives (EMD). It is reported that expression of cementum attachment protein (CAP) and cementum protein-23 (CP-23) has been detected in whole DF stimulated by EMD and in cultured DFPCs stimulated by EMD or BMP2/7 [10]. A recent study demonstrated the role of discoidin domain receptor 2 (Ddr2) during development of periodontium tissue and tooth root. Ddr2-LacZ knock-in mice showed the abundant expression of Ddr2 in DF. Widened PDL space and interradicular alveolar bone defects along with abnormal collagen content within the PDL were observed in Ddr2-null mice [83].

DFPCs differentiate into periodontal specific lineages in a growth factor dependent way [84]. DFPCs cultured in the induction medium containing recombinant human fibroblast growth factor-2 (rhFGF-2) or recombinant human cementum protein-1 (rhCEMP-1) presented higher expression of fibroblast or cementoblast related genes. Another cytokine, F-spondin, is reported to suppress the differentiation of DFPCs into PDL by inhibiting TGF-β. F-spondin decreased the expression of periostin and Col1 in DF. Thus, it may function to maintain the immature phenotype of the DF [85].

## 3. DFPCs in Tissue Engineering

### 3.1. The Potential of DFPCs in Dental Tissue Engineering

#### 3.1.1. Craniofacial Bone Regeneration

A number of studies have demonstrated that DFPCs participate in the formation of craniofacial bone. After being transplanted into dorsum of immunodeficient mice, DFPCs formed bone-like hard tissue, which makes DFPCs-based therapy feasible in the treatment of calvaria and alveolar bone defect [74]. Tsuchiya et al. first applied DFPCs to regenerate bone tissue in the calvaria defect. They constructed a critical size parietal defect in porcine and filled it with cell pellet [86]. After four weeks, hard tissue was observed in the defect sites, and the bone area generated in DFPCs transplantation group was more extensive. Subsequently, DFPCs were implanted in skull defects of rats, which showed approximately 50% woven bone formation after eight weeks [87]. These studies demonstrated a capacity of craniofacial bone regeneration of DFPCs. On the other hand, DFPCs, as seeding cells of bio-hybrid implants, were transplanted into a bony hole of alveolar bone in a tooth-loss model. After seven weeks, the bio-hybrid implant restored physiological functions and regenerated the severe bone defect [88]. Noteworthy, it was not certain enough to obtain effective tissue repairment relying on cell differentiation singly. The scaffold of bio-hybrid implants above conducted DFPCs adhesion and proliferation. It was considered as a candidate for artificial organ building. It is reported that dynamic culture conditions enhanced the migration of DFPCs into the inner part of the scaffolds and contribute to higher tissue ingrowth after being implanted subcutaneously in vivo [78]. Moreover, physiological status of DFPCs plays a critical role in osteogenic differentiation, and DFPCs derived from inflammatory environment displayed less hard tissue formation than normal cells [74]. It is notable that the passage of DFPCs also has an effect on tissue regeneration. DFPCs at late passage could lose their osteogenic ability and only form connective tissues [89].

#### 3.1.2. Periodontal Tissue Regeneration

Periodontitis is a common prevalent oral disease characterized by excessive host immune response, and results in inflammation and destruction of tooth supporting tissues [90]. Traditional clinical therapies can only control the progression of periodontitis, which are limited in restoring the complete periodontal tissue. MSC-based tissue regeneration is a novel trend for the treatment for periodontitis. As precursor cells participating in periodontal tissue formation during tooth development, DFPCs have been considered as the most attractive candidates for PDL regeneration. By transferring DFPCs into the carrier chamber and seeding into renal capsules, Wu et al. found that DFPCs can form a large mass of cementum-like tissue distinct from the original dentin [91]. In addition, subcutaneous transplantation of DFPCs with dentin matrix in nude mice for 6 weeks resulted in the formation of cementum-like tissue with embedded fibers and PDL-like tissue along with blood vessels [92]. Furthermore, DFPCs sheets were implanted in two-wall intrabony defects of canine experimental periodontitis. After one month, a complex cementum-PDL structure was observed in the defect area, implying the potential of DFPCs to regenerate complete periodontal tissue [93]. Besides structural recovery, periodontal tissue regenerated by DFPCs can also recover the physiological functions, including the ability to perceive noxious mechanical and chemical stimulation, highlighting a significant advancement of DFPCs in periodontal regeneration [88]. Additionally, cementum and PDL-like tissue formation were markedly promoted when DFPCs were pre-exposed to HERS cells via epithelial-mesenchymal interactions, indicating an appropriate stimulation from HERS can induce the differentiation and immigration of DFPCs [94,95]. Although periodontal ligament stem cells (PDLSCs) have been proven to regenerate periodontal tissue as well, some comparative studies found the distinct regeneration capacity between DFPCs and PDLSCs. Proteomic analysis revealed that there were 32 differentially expressed proteins between DFPCs and PDLSCs [96]. After being transplanted into periodontal defects, DFPCs exhibited a stronger capacity for regeneration of cementum and periodontal attachment than PDLSCs [92]. It was speculated that DFPCs harbored a better periodontal regeneration ability due to participation of the extracellular matrix, yet the regulatory mechanisms need further investigation.

#### 3.1.3. Root-Like Tissue Regeneration

Tooth loss is commonly caused by periodontal diseases, trauma, endodontic complications and other diseases. The applications of dental implants are widely recommended in clinical practice. However, implants cannot acquire a physiological movement and defend overload stress due to the lack of the periodontal tissue, which limits the effect of the therapy [97]. Apart from bone and cementum-PDL structure, DFPCs can also regenerate dentin-pulp like tissue, which makes it possible to regenerate an integrated tooth root. Yang et al. seeded DFPCs to biological scaffolds and implanted them into nude mice subcutaneously [98]. Eight weeks later, they found cementum-PDL complex, which consists of cementum, periodontal ligament fibers and blood vessels outside the scaffold. Meanwhile, they also observed dentin-pulp like tissue reconstruction, including dentinal tubules, pre-dentin, polarizing odontoblast-like structures and collagen fibers. This finding brings hope to root regeneration therapy. DFPCs-based root regeneration makes it possible to form bio-root, which is closer to natural root. After that, DFPCs combined with biological scaffolds were transplanted into alveolar fossa, omental pockets and cranial fossa. Interestingly, although mineralized matrix was observed in omental pockets and cranial fossa, root-like tissues were only formed in alveolar fossa. This study implies that the microenvironment of the alveolar fossa is suitable for tooth root construction when DFPCs are applied [99]. A bio-root complex was constructed through combining DFPCs with treated dentin matrix (TDM) scaffold. Using computer aided design (CAD), this bio-root complex not only successfully regenerated root-like structure but also performed masticatory function and remained stable for at least three months after crown restoration [100].

#### 3.1.4. Providing a Favorable Microenvironment

In physiological conditions, MSCs secrete abundant factors and cytokines, such as TGF-β, hepatic growth factor (HGF), prostaglandin E2 (PGE2), interleukin-10 (IL-10), nitric oxide (NO), indolamine2, 3-dioxygenase (IDO), heme oxygenase-1 (HO-1) and human leukocyte antigen-G (HLA-G). These factors mediate communication between MSCs and other cells, which play a pivotal role in the immunomodulatory function of MSCs [101]. As one of the MSCs in dental region, DFPCs also express various soluble factors. A protein array showed that there were 42 differentially expressed proteins in the conditioned medium of DFPCs (DFPCs-CM) compared with normal medium, including growth factors, cytokines, chemokines, matricellular proteins and transmembrane proteins [102]. These factors play a critical role in stemness maintaining and immunomodulation by constructing a specific microenvironment. In this context, DFPCs can provide a beneficial environment and act on other cells through a paracrine manner in MSC-based tissue engineering. Therefore, it is advisable to explore immunomodulation of DFPCs in oral diseases [103].

Pulpitis is a common inflammatory disease leading to irreversible pulp destruction and necrosis [104]. DFPCs attenuated LPS-induced inflammatory dental pulp cells (DPCs) through paracrine mechanisms. DFPCs-CM downregulated the extracellular regulated protein kinases 1/2 (ERK1/2) and nuclear factor kappa-B (NF-κB) signaling pathways to suppress IL-1β, IL-6. Furthermore, DFPCs-CM enhanced the capacity of proliferation, migration and odontogenesis of inflammatory DPCs. Capped with an aseptic gelatin sponge soaked in DFSCs-CM, pulp tissue showed a relieved inflammatory infiltration and increased Runx2 expression in odontoblast-like cells near the injured site. These findings bring up a novel therapy using DFSCs-CM to attenuate excessive inflammation and preserve pulp vitality.

Additionally, DFPCs could provide a favorable microenvironment for PDLSCs regeneration. Liu et al. harvested PDLSCs from healthy individual (HPDLSCs) and periodontitis patients (PPDLSCs). They found that PPDLSCs had weaker pluripotency and differentiation capacity compared to HPDLSCs, verifying that microenvironment in periodontitis has an adverse effect on PDLSCs, which may postpone periodontal regeneration. Nevertheless, when pretreated PPDLSCs with DFPCs-CM and then transplanting them into immunodeficient mice, they found an improvement of proliferation and differentiation of PPDLSCs. In addition, DFPCs promoted the formation of cell layers and extracellular matrix in PDLSCs cell sheets, and PDLSCs pretreated with DFPCs-CM improved periodontal regeneration in vivo [105]. DFPCs may provide a beneficial microenvironment to restore the biological impairment of PPDLSCs through a paracrine manner and ultimately promote periodontal regeneration of PDLSCs, yet the underlying mechanisms remain to be explored.

CNCCs are a clump of multipotent embryonic stem cells arising from the neural folds of the developing embryo, and can differentiate into various dental tissues, such as dental papilla and dental follicles. Therefore, CNCCs are essential for tooth morphogenesis. However, CNCCs tend to spontaneously differentiate into smooth muscle or osteoblast lineages due to their heterogeneity, which significantly limits clinical application of CNCCs. Wen et al. isolated p75NTR positive (p75^+^) CNCCs by fluorescence activated cell sorter, and then incubated them in DFPCs-CM combined with dentin non-collagenous proteins (dNCPs) [106]. As a result, the morphological features of p75^+^ CNCCs have been altered, along with increased calcified nodule formation and higher expression of cementoblast lineage related markers when treated with dNCPs/DFPCs-CM. Specific cellular and acellular components usually provide a particular microenvironment and govern stem cell fate [107]. These results indicated that dNCPs/DFPCs-CM may direct the differentiation of dental stem cells into cementoblast lineage.

#### 3.1.5. DFPCs and Scaffolds

Stem cell based regenerative medicine has been acknowledged as a hot topic to recover the damaged and lost tissues and regain the function. Although stem cell sheets provide a living microenvironment for the seeded cells, the low survival rate of implanted cells and failure of an integrated morphology reconstruction are major challenges in the development of cellular transplantation. Besides multipotent seeding cells, biomaterial scaffolds are crucial for tissue regeneration. An ideal scaffold not only maintains a long-term mechanical strength, but also provides a bioactive microenvironment to mimic the native extracellular matrix. Currently, a variety of scaffolds combined with DFPCs have been applied to tooth engineering (Table 1).

Ceramic scaffolds, including hydroxylapatite (HA) and tricalcium phosphate (TCP), were the most extensively applied materials in DFPCs regeneration because of their biocompatibility properties. HA powder was first applied with DFPCs in 2002 [108]. Then porous HA ceramic discs were proved to induce the osteogenesis of DFPCs [9]. After that, a variety of scaffolds, such as HA/collagen-gel, HA-coated dental implant and HA/TCP, showed favorable for DFPCs adhesion and proliferation abilities as well as dental tissue differentiation [74,88,109]. As their chemical property and physical ingredient are similar to native bone, ceramic scaffolds can replicate a unique environment for DFPCs fate.

Furthermore, natural polymers and native structures were widely used due to their availability and convenience. Ceramic bovine bone (CBB) was one of the easiest native structure scaffolds [111]. A cementum-PDL complex was observed when transplanting DFPCs combined with CBB. dNCPs consist of a mixture of proteins extracted from dentin, including glycoproteins/sialoproteins, phosphoproteins, proteoglycans and growth factors. Studies suggested that dNCPs scaffold could stimulate DFPCs to differentiate into cementoblast lineages [91]. TDM is a dentin-based decellularization scaffold and generated by sequential demineralization [99]. It has been reported that TDM has a good bioactivity and biocompatibility, and can release key factors to induce tooth development. The release of osteogenic proteins induces the osteogenic differentiation of DFPCs, which accelerates bone defects recovery and cementum-PDL complex regeneration. More importantly, TDM can promote odontogenesis of DFPCs due to odontogenic-related proteins. Thus, the combination of DFPCs and TDM successfully regenerated cementum-PDL complex and dentin-pulp like tissue, making it possible to regenerate complete bio-root [110].

More recently, the development of synthetic materials enlighten studies related to transplanted scaffolds. Graphene-oxide (GO) and its derivatives, fluoride nanosilicate platelets (NS+F) and titanium implants with HA (TiHA) were applied in DFPCs-based regeneration in vitro. The study found that GO was able to support cellular attachment, proliferation and differentiation of dental stem cells [115]. Both GO and nitrogen-doped graphene (N-Gr) have low cytotoxic effects and injury of oxide stress for DFPCs, indicating they are valuable candidates for DFPCs scaffolds [114]. In addition, NS+F revealed a similar effect on providing a suitable microenvironment and enhancing DFPCs osteogenesis [113]. Moreover, TiHA can induce osteogenic differentiation of DFPCs even in the absence of exogenous factors owing to their chemical and topographical properties [112]. As the ingredient is specific, synthetic materials seem to be more controllable and predictable, resulting in repeatable results.

### 3.2. Application in Other Diseases

#### 3.2.1. Nervous Tissue Regeneration

Apart from dental tissues, DFPCs have been proven as a candidate for the treatment of spinal cord injury (SCI). SCI is a severe disease leading to a series of impairments in sensory, motor and autonomic functions. The pathological process of SCI is complicated, characterized by tissue damage in the acute phase and a cascade of secondary necrotic of neurons and astrocytes, resulting in an irreversible loss of axons and demyelination [116,117]. By far, the effect of traditional therapies, such as supportive treatment and pharmacotherapy, are still unsatisfactory [118]. Owing to DFPCs originating from the cranial neural crest and expressing some neurogenic membrane markers, such as nestin and tubulin β III, it was supposed that DFPCs harbored the potential in neural regeneration [119]. Li et al. transplanted human DFPCs (hDFPCs) to restore the defect in rat spinal cord and demonstrated that implanted hDFPCs expressed oligodendrogenic lineage maker oligodendrocyte lineage transcription factor 2 (Olig2) in vivo, therefore contributing to remyelination [120]. Further studies revealed the mechanisms of DFPCs in SCI repair. First, DFPCs function to suppress inflammatory response in the acute phase through inhibiting the expression of IL-1β. They also reduce secondary hemorrhagic necrosis via downregulating the expression of sulfonylurea receptor 1 (SUR-1). Secondly, DFPCs inhibit the activity of ras homolog gene family member A (RhoA), which is induced by injury and restrained neurite regeneration. Lastly, survived DFPCs are able to differentiate into mature neurons and oligodendrocytes but not astrocytes, avoiding the formation of glial scar [121]. In sum, DFPCs may have a potential for neural differentiation, and DFPCs-based therapy brings a new strategy for diseases related to nervous system.

#### 3.2.2. Therapy of Autoimmune and Inflammatory Diseases

MSCs-based therapy is promising to modulate the immune system and is widely used in preclinical treatment studies of several autoimmune disorders and inflammatory diseases [103]. Specifically, MSCs have an effect on both innate and adaptive immunity by regulating immune cells, including T cells, B cells, natural killer cells, monocytes/macrophages, dendritic cells and neutrophils [122]. A large variety of soluble factors secreted by MSCs, such as TGF-β1, IDO and PGE2 can reduce immunoreaction [123]. In turn, interferon γ (IFN-γ) produced by T cells could upregulate adhesion molecules of MSCs, such as programmed death ligand 1 (PDL-1) and vascular cell adhesion molecule-1, and increase the immunomodulation capacity of MSCs [124].

Recently, MSCs reserved in dental and oral tissue, were successfully isolated, which sparked the application of dental derived MSCs. The easily accessibility and less ethical issues encourage DFPCs application in autoimmune diseases. Ulusoy et al. used DFPCs to administrate myasthenia gravis (MG) in 2015 [125]. MG is a T cell-associated autoimmune disease. Acetylcholine receptor (AChR) antibodies induce the dysfunction of neuromuscular junction (NMJ), resulting in fluctuating muscle weakness [126]. Currently, steroids, azathioprine and other cytotoxic drugs, are applied to suppress global immunoreaction in MG patients, but traditional pharmacotherapy is accompanied by substantial side effects [126]. Therefore, it is urgent to find novel therapeutic treatment strategies. After DFPCs inoculation, mice with MG showed decreased incidence and clinical symptoms, accompanied by lower serum levels of anti-IgG1, IgG2b and IgG3. Moreover, DFPCs inhibited the proliferation of lymph node cells, and suppressed lymphocyte responses to IL-6 and IL-12. Strikingly, cytokines secreted by T cells and B cells were not affected, indicating DFPCs mainly suppressed the innate immune system in MG. Overall, DFPCs therapy is beneficial to ameliorate MG through a distinct regulatory effect on immunity.

DFPCs have also been applied in the treatment of asthma. Asthma is a chronic inflammatory disease mediated by T helper type 2 cells (Th2). Allergen exposure could lead to an inflammation in airway and epithelial damage. In this process, antigen is presented to naïve T lymphocytes by dendritic cells, and then Th2 is polarized [127]. Meanwhile, B cells produce IgE antibodies to bind with mast cells, which could recruit immune cells when re-exposed to allergens [128]. However, pharmacotherapy of corticosteroids and glucocorticoids may bring side effects, and allergen specific immunotherapy needs long time and has a risk of anaphylaxis. Therefore, a short-term and safe approach is needed to modulate immune system with less side effects. To figure out the effect of DFPCs in asthma, peripheral blood mononuclear cells (PBMCs) of asthmatic patients were isolated and co-cultured with DFPCs in vitro. They found that DFPCs inhibited proliferation of CD4^+^ T lymphocytes by increasing CD4^+^CD25^+^ regulatory T cell (Treg) amounts [129]. Additionally, IFN-γ stimulation enhanced the potential capacity of immunosuppression of DFPCs. Subsequently, they found DFPCs inhibited Th2 polarization by decreasing IL-4 cytokine levels and reduced the costimulatory activation of monocytes [130]. These results implied that DFPCs can enhance immune tolerance in allergic asthma, suggesting DFPCs would be an ideal therapeutic drug for inflammation diseases. Nonetheless, these results mentioned above are based on in vitro experiments. Considering a complicated interaction in vivo, extensive analysis using animal models is needed in the future.

## 4. Future Perspective

DFPCs are an important type of dental stem cells originating from dental follicles and play a critical role during tooth development. During tooth eruption, DFPCs can provide a traction power and participate in construction of an eruption pathway via mediating alveolar bone formation and resorption. Furthermore, DFPCs contribute to the development and maintenance of an periodontal attachment apparatus, which is beneficial to perceive mechanical stress and exert physiological function of the tooth. With high pluripotency, DFPCs can differentiate into osteoblasts, cementoblasts, adipocytes and PDL cells as well as neuronal cells, implying potential applications in tissue engineering. In the past decades, stem cell-based therapy brings a hope to regenerative medicine, but difficulties in cellular acquiring and ethical issues limit the development of this therapy. DFPCs can be harvested from discarded tooth conveniently and noninvasively, making them a promising source of stem cells in regenerative medicine.

In tissue engineering, a feasible therapeutic tool involves stem cells, scaffolds and specific factors. Although progress has been made in understanding the remarkable tissue regeneration ability of DFPCs, there are still several limitations, which need further investigations. First, derived from dental follicle, DFPCs were considered as a heterogeneous population, which showed variational phenotypes and differentiative capacities [111]. Heterogeneity disturbs directional differentiation of DFPCs and reduces efficiency for tissue regeneration. Thus, future studies need to thoroughly identify subpopulations of DFPCs. The rigorous single-cell technology will be hopeful to analyze new markers to distinguish each subgroup, which may accelerate generating new transgenic mouse models to reveal the distinct characteristics of each subgroup. Second, current research mainly chose dorsum and omenta transplantations in animal experiments for tissue regeneration. However, root-like tissues, developed from DFPCs and TDM complex, can only be regenerated in the alveolar fossa, implying the significance of microenvironment in cellular differentiation [99]. Studies using in situ transplantations are important to understand the differentiation trait of DFPCs. Third, DFPCs have been reported to restore mineralized tissue in calvaria and alveolar bone defects with integrated morphology, but it is urgent to assess the physical characteristics of newly formed tissues. Notwithstanding, the masticatory function of bio-root complex show a success after three months usage; a long-term observation is needed to evaluate its function [100]. Lastly, an ideal scaffold is of vital importance in DFPCs-based tissue engineering. The rapid development of novel synthetic materials and advanced preparation technology will push a perfect implanting approach forward.

## Figures and Tables

**Table 1:** Summary of the recent evidence of DFPCs with scaffolds in regeneration medicine.

Scaffolds	Tissue Regeneration	Origin	Transplantation Model	Reference
hydroxyapatite (HA) powder	Fibrous tissues and cementum-like matrix	Bovine	SCID mice subcutaneous pockets	Handa et al. 2002 [108]
HA-coated dental implant	Bone-like and PDL tissues	Murine	Mice tooth-loss model	Oshima et al. 2014 [88]
HA ceramic discs	Cement/woven bone-like tissues	Human	Immunocompromised rats subcutaneous pockets	Yagyuu et al. 2010 [9]
HA/collagen-gel	Acellular cementum-like tissues	Human	SCID mice subcutaneous pockets	Shinagawa-Ohama et al. 2016 [109]
HA/tricalcium phosphate particles	Bone-like tissues	Human	Immunodeficient mice subcutaneous pockets	Um et al. 2018 [74]
Collagen nanohydroxyapatite/phosphoserine biocomposite cryogel	Bone-like tissues	Human	Immunodeficient mice subcutaneous pockets	Salgado et al. 2020 [78]
Treated dentin matrix (TDM)	Periodontal-like tissues	Canine	Canine one-wall periodontal intrabony defects	Yang et al. 2019 [110]
TDM	Root-like tissues	Rat	Rats alveolar fossa	Guo et al. 2012 [99]
TDM	Dentin-like tissues	Human	Immunodeficient mice subcutaneous pockets	Tian et al. 2015 [96]
TDM	Periodontal-like tissues	Human	Nude mice subcutaneous pockets	Guo et al. 2013 [92]
TDM	Root-like tissues	Human	Immunodeficient mice subcutaneous pockets	Yang et al. 2012 [98]
Extracellular matrix	Bone-like tissues	Porcine	Immunocompromised rats critical size parietal defect	Tsuchiya et al. 2010 [86]
Dentin non-collagenous proteins (dNCPs)	Cementum-like tissues	Rat	Rats renal capsules	Wu et al. 2008 [91]
Ceramic bovine bone	Cementum-PDL complex	Human	Nude mice subcutaneous pockets	Guo et al. 2012 [111]
Titanium implants with hydroxyapatite (TiHA), with silicatitanate (TiSiO2)	Enhanced osteogenic differentiation capabilities	Human	In vitro	Lucaciu et al. 2015 [112]
Fuoride nanosilicate platelets (NS+F)	Enhanced osteogenic differentiation capabilities	Human	In vitro	Veernala et al. 2019 [113]
Graphene-oxide (GO), Thermally reduced graphene oxide (TRGO), Nitrogen-doped graphene (N-Gr)	low levels of cytotoxicity and mitochondria induced damage	Human	In vitro	Olteanu et al. 2015 [114]

## Data Availability

Not applicable.
